# Should singleton birth weight standards be applied to identify small-for-gestational age twins?: analysis of a retrospective cohort study

**DOI:** 10.1186/s12884-021-03907-1

**Published:** 2021-06-25

**Authors:** Dongxin Lin, Jiaming Rao, Dazhi Fan, Zheng Huang, Zixing Zhou, Gengdong Chen, Pengsheng Li, Xiafen Lu, Demei Lu, Huishan Zhang, Caihong Luo, Xiaoling Guo, Zhengping Liu

**Affiliations:** 1 Foshan Institute of Fetal Medicine, Southern Medical University Affiliated Maternal & Child Health Hospital of Foshan, 11 Renminxi Road, Guangdong 528000 Foshan, China; 2 Department of Obstetrics, Southern Medical University Affiliated Maternal & Child Health Hospital of Foshan, 528000 Foshan, Guangdong China; 3 The First Affiliated Hospital of Guangdong Pharmaceutical University, 510030 Guangzhou, Guangdong China

**Keywords:** Twins, Birth weight percentiles, Small for gestational age, Neonatal outcomes

## Abstract

**Background:**

Twin birth weight percentiles are less popular in clinical management among twin pregnancies compared with singleton ones in China. This study aimed to compare the incidence and neonatal outcomes of small for gestational age (SGA) twins between the use of singleton and twin birth weight percentiles.

**Methods:**

This was a retrospective cohort study of 3,027 pregnancies with liveborn twin pairs at gestational age of > 28 weeks. The newborns were categorized as SGA when a birthweight was less than the 10th percentile based on the singleton and twin references derived from Chinese population. Logistic regression models with generalized estimated equation (GEE) were utilized to evaluate the association between SGA twins and neonatal outcomes including neonatal unit admission, neonatal jaundice, neonatal respiratory distress (NRDS), neonatal asphyxia, ventilator support, hypoxic ischemic encephalopathy (HIE), bronchopulmonary dysplasia (BPD), necrotizing enterocolitis (NEC), intracranial hemorrhage (ICH), culture-proven sepsis, neonatal death within 28 days after birth as well as the composite outcome.

**Results:**

The incidence of SGA was 33.1 % based on the singleton reference and 7.3 % based on the twin reference. Both of SGA newborns defined by the singleton and twin references were associated with increases in neonatal unit admission, neonatal jaundice and ventilator support. In addition, SGA newborns defined by the twin reference were associated with increased rates of BPD (aOR, 2.61; 95 % CI: 1.18–5.78) as well as the severe composite outcome (aOR, 1.93; 95 % CI: 1.07–3.47).

**Conclusions:**

The use of singleton birth weight percentiles may result in misdiagnosed SGA newborns in twin gestations and the twin birth weight percentiles would be more useful to identify those who are at risk of adverse outcomes.

**Supplementary Information:**

The online version contains supplementary material available at 10.1186/s12884-021-03907-1.

## Background

Birth weight and fetal weight are common indicators of fetal growth. However, the terminology for newborns with abnormal growth is inconsistent. In the updated American College of Obstetricians and Gynecologist (ACOG) Practice Bulletin, fetal growth restriction (FGR) is defined based on a sonographic estimated fetal weight (EFW) less than 10th percentile for gestational age while small for gestational age (SGA) is defined based on a birth weight less than 10th percentile for gestational age [1]. FGR or SGA increases the risks of perinatal morbidity and mortality [2–4]. Furthermore, these categories of children are also at risks of long-term complications such as neurodevelopmental delay, adult obesity and metabolic diseases [5–8].

Over the last decades, the incidence of twin pregnancies has been rising mainly due to the significantly increased use of assisted reproductive technology (ART) and the delayed childbearing age [9–11]. Compared with singletons, twin pregnancies have higher risks of FGR or SGA. Therefore, correctly identifying those fetuses with abnormal growth is of great clinical significance for improving prediction of adverse outcomes and perinatal health care for twins. In fact, there remains controversy as to which reference should be used to classify SGA twins, mainly due to the geographic and ethnic disparities in fetal growth pattern. Twin-specific birth weight percentiles for gestational age have been established and updated from regional or national databases [12–20]. However, these tools seem not popular because of lack of linking data to perinatal outcomes. In contrast, the singleton references were commonly used in clinical practice and research work [21–23].

In this study, we compared the prevalence of SGA twins defined by the singleton-based and the twin-based birth weight percentiles and their associations with neonatal morbidity and mortality.

## Methods

### Study design

The retrospective cohort study included twin deliveries at a tertiary hospital in Foshan, China, from January 2012 to December 2020. This study was approved by the ethics committee of Southern Medical University Affiliated Maternal & Child Health Hospital of Foshan (FSFY-2,019,042,006) and the ethics committee endorsed this research to waive the informed consent of the patients due to the retrospective design.

### Inclusion and exclusion criteria

The inclusion criteria for current study were pregnancies with both living born fetuses at gestational age beyond 28 weeks. Pregnancies with congenital anomalies, twin-to-twin transfusion (TTTS), monoamniotic twins, unknown chorionicity, intrauterine death and gestational age at delivery < 28 weeks were excluded for analysis.

### Data collection

All electric medical records of eligible pregnancies were systematically reviewed to obtain maternal and neonatal Information. Data on maternal age, parity, use of assisted reproductive technology, chorionicity, fetal gender, gestational complications (gestational diabetes mellitus, idiopathic jaundice of pregnancy and hypertensive disorder of pregnancy), maternal infections (syphilis and hepatitis B) and pre-existing hypertension and diabetes mellitus was collected. In the present study, the chorionicity was determined by sonographic examination and was confirmed by placental pathologic findings after birth, if available. The gestational age was calculated from the date of embryo transfer for IVF/ICSI pregnancies (+ 14 days) and based on the last menstrual period for spontaneous pregnancies and was confirmed by sonography in the first trimester. HDPs included gestational hypertension and preeclampsia (PE), which were diagnosed based on the criteria developed by ACOG [24]. A diagnosis of gestational diabetes mellitus (GDM) was made by oral 75 g glucose tolerance test (OGTT) between 24 and 28 weeks (fasting plasma glucose ≥ 5.1 mmol/l or 1-hour plasma glucose ≥ 10.0 mmol/l or 2-hour plasma glucose ≥ 8.5 mmol/l). SGA was defined when a birthweight was less than the 10th percentile. We utilized population-based birthweight percentiles in Chinese singletons and twins [15, 25]. These references were chosen because of their large size of study population and adjustment for fetal sex.

### Outcomes of interest

The primary outcome was neonatal unit admission. The secondary outcomes included neonatal jaundice, neonatal respiratory distress (NRDS), neonatal asphyxia, ventilator support, hypoxic ischemic encephalopathy (HIE), bronchopulmonary dysplasia (BPD), necrotizing enterocolitis (NEC), intracranial hemorrhage (ICH), culture-proven sepsis and neonatal death within 28 days after birth. We also defined a composite outcome as any occurrence of HIE, NEC, ICH, BPD, sepsis as well as neonatal death.

### Statistical analysis

The baseline characteristics of the included pregnancies were reported as mean ± standard deviation (SD) or frequency with its percentage, where appropriate. When analyzing the neonatal outcomes, the unit of analysis was the newborn. In this regard, logistic regression analyses were performed using a generalized estimating equation (GEE) approach to address the intertwin correlation. The adjusted odds ratios (ORs) and 95 % confidence intervals (CIs) were calculated by performing multivariable models controlled for gestational age at delivery, nulliparity, maternal age, chorionicity, use of ART, GDM and pre-eclampsia. We also performed sensitivity analysis after excluding women with complications during pregnancy (ICP, HDPs, GDM), pre-existing hypertension or diabetes mellitus and maternal infection (syphilis and hepatitis B). All two tailed *P*-values < 0.05 were considered statistical significance. The statistical analyses were performed using Stata, version 15.1.

## Results

The baseline characteristics of the included pregnancies were shown in Table 1. During the study period, 3,027 twin pregnancies with 6,054 newborns were eligible for the study. The maternal age was 30.8 ± 4.6 (range: 17–52). Over half of the included pregnancies were nulliparity (67.2 %) and ART conceived (65.4 %). Monochorionic twins accounted for 17.9 %. The proportion of fetal gender combination was 34.8 %, 28.9 and 36.3 % for male-male, female-female and male-female, respectively. The incidence of hypertensive disorders of pregnancy was 11.3 % and that of GDM was 20.3 %. The incidences of idiopathic jaundice of pregnancy, pre-existing diabetes mellitus, pre-existing hypertension and syphilis were low (2.7 %, 1.0 %, 0.8 and 0.3 %, respectively). The incidence of Hepatitis B was 7.7 %. The vast majority of included pregnancies (98.3 %) underwent caesarean section. The gestational age at delivery was 35.8 ± 1.8 weeks. The incidence of SGA fetuses was 33.1 % based on the singleton standards and 7.3 % based on twin standards. The incidence of LGA fetuses was 0.3 and 4.8 % based on singleton and twin standards, respectively.

**Table 1:** Baseline characteristics of included pregnancies (*N* = 3027)

Variables	
Maternal age	30.8 ± 4.6 (range: 17–52)
Nulliparity	2034 (67.2)
ART conceived pregnancies	1981 (65.4)
Monochorionic twins	542 (17.9)
Fetal sex	
Male-male	1053 (34.8)
Female-female	874 (28.9)
Male-female	1100 (36.3)
Preexisting diabetes mellitus	29 (1.0)
Preexisting hypertension	24 (0.8)
Syphilis	8 (0.3)
Hepatitis B	234 (7.7)
Hypertensive disorders of pregnancy	342 (11.3)
Gestational diabetes mellitus	615 (20.3)
Idiopathic jaundice of pregnancy	81 (2.7)
Caesarean section	2974 (98.3)
Gestational age at delivery	35.8 ± 1.8
Classification of twin birth weight	
Singleton standards	
Small for gestational age	2002 (33.1)
Appropriate for gestational age	4036 (66.7)
Large for gestational age	16 (0.3)
Twin standards	
Small for gestational age	443 (7.3)
Appropriate for gestational age	5322 (87.9)
Large for gestational age	289 (4.8)

*ART* assisted reproductive technology

In the multivariable analyses, as shown in Table 2, we found that the SGA newborns defined based on singleton standards were associated with increased odds ratios of neonatal unit admission (aOR, 2.11; 95 % CI: 1.84–2.41), neonatal jaundice (aOR, 1.99; 95 % CI: 1.73–2.29) and ventilator support (aOR, 1.45; 95 % CI: 1.09–1.91) but were not associated with any other morbidities and mortality.

**Table 2:** Analysis of association between SGA twin newborns and neonatal outcomes based on singleton birthweight reference

Outcomes	Non-SGA (*n* = 4,052)	SGA (*n* = 2,002)	Unadjusted OR (95 % CI)	*P*-value	Adjusted OR (95 % CI)^a^	*P*-value
Neonatal unit admission	1629 (40.2)	878 (43.9)	1.31 (1.20–1.44)	< 0.001	2.11 (1.84–2.41)	< 0.001
Neonatal jaundice	1191 (29.4)	627 (31.3)	1.27 (1.15–1.40)	< 0.001	1.99 (1.73–2.29)	< 0.001
NRDS	413 (10.2)	120 (6.0)	0.82 (0.72–0.94)	0.004	1.11 (0.88–1.41)	0.376
Neonatal asphyxia	108 (2.7)	34 (1.7)	0.70 (0.49–0.99)	0.045	0.96 (0.63–1.44)	0.828
Ventilator support	351 (8.7)	110 (5.5)	0.78 (0.65–0.94)	0.008	1.45 (1.09–1.91)	0.010
HIE	12 (0.3)	6 (0.3)	1.01 (0.38–2.70)	0.980	1.04 (0.38–2.86)	0.934
ICH	17 (0.4)	8 (0.4)	0.97 (0.42–2.26)	0.941	1.09 (0.46–2.61)	0.846
Sepsis	50 (1.2)	18 (0.9)	0.76 (0.44–1.30)	0.312	1.20 (0.67–2.14)	0.533
BPD	50 (1.2)	14 (0.7)	0.66 (0.37–1.17)	0.157	1.61 (0.80–3.21)	0.180
Neonatal death	16 (0.4)	8 (0.4)	1.01 (0.43–2.37)	0.982	1.39 (0.56–3.45)	0.475
Severe composite outcome	101 (2.5)	28 (1.4)	0.67 (0.45-1.00)	0.051	1.34 (0.83–2.16)	0.230

^a^Adjusted for gestational age at delivery, nulliparity, maternal age, chorionicity, use of ART, PE and GDM. *NRDS*, neonatal respiratory distress syndrome; *HIE* hypoxic ischemic encephalopathy, *ICH* intracranial hemorrhage, *BPD* bronchopulmonary dysplasia, *OR* odds ratio, *ART* assisted reproductive technology, *PE* pre-eclampsia, *GDM*, gestational diabetes mellitus

Based on the twin standards, similarly, SGA newborns were associated with neonatal unit admission (aOR, 7.72; 95 % CI: 6.01–9.91), neonatal jaundice (aOR, 4.09; 95 % CI: 3.30–5.09) and ventilator support (aOR, 1.94; 95 % CI: 1.32–2.85). In addition, they were associated with increased odds ratios of BPD (aOR, 2.61; 95 % CI: 1.18–5.78) as well as the severe composite outcome (aOR, 1.93; 95 % CI: 1.07–3.47) (Table 3).

**Table 3:** Analysis of association between SGA twin newborns and neonatal outcomes based on twin birthweight reference

Outcomes	Non-SGA (*n* = 5,611)	SGA (*n* = 443)	Unadjusted OR (95 % CI)	*P*-value	Adjusted OR (95 % CI)^*^	*P*-value
Neonatal unit admission	2153 (38.4)	354 (79.9)	3.68 (3.13–4.34)	< 0.001	7.72 (6.01–9.91)	< 0.001
Neonatal jaundice	1554 (27.7)	264 (59.6)	2.58 (2.20–3.03)	< 0.001	4.09 (3.30–5.09)	< 0.001
NRDS	475 (8.5)	58 (13.1)	1.05 (0.85–1.30)	0.674	1.25 (0.90–1.75)	0.187
Neonatal asphyxia	128 (2.3)	14 (3.2)	1.24 (0.75–2.07)	0.397	1.22 (0.69–2.13)	0.498
Ventilator support	410 (7.3)	51 (11.5)	1.35 (1.05–1.75)	0.021	1.94 (1.32–2.85)	0.001
HIE	15 (0.3)	3 (0.7)	2.54 (0.73–8.82)	0.141	2.06 (0.57–7.36)	0.268
ICH	21 (0.4)	4 (0.9)	2.50 (0.86–7.22)	0.091	2.05 (0.68–6.13)	0.201
Sepsis	60 (1.1)	8 (1.8)	1.60 (0.75–3.41)	0.219	1.54 (0.71–3.31)	0.273
BPD	54 (1.0)	10 (2.3)	2.33 (1.20–4.52)	0.013	2.61 (1.18–5.78)	0.018
Neonatal death	19 (0.3)	5 (1.1)	3.00 (1.08–8.28)	0.034	2.34 (0.82–6.71)	0.113
Severe composite outcome	113 (2.0)	16 (3.6)	1.74 (1.04–2.91)	0.035	1.93 (1.07–3.47)	0.028

^a^Adjusted for gestational age at delivery, nulliparity, maternal age, chorionicity, use of ART, PE and GDM. *NRDS* neonatal respiratory distress syndrome, *HIE*, hypoxic ischemic encephalopathy, *ICH* intracranial hemorrhage, *BPD* bronchopulmonary dysplasia, *OR* odds ratio; *ART* assisted reproductive technology, *PE* pre-eclampsia, *GDM* gestational diabetes mellitus

After excluding women with complications during pregnancy (ICP, HDPs and GDM), pre-existing hypertension or diabetes mellitus or maternal infections, SGA newborns, irrespective of birth weight percentiles, were associated with increased incidences of neonatal unit admission, neonatal jaundice and ventilator support. However, the SGA newborns did not have significantly increased incidences of severe morbidities and mortality. The results were supplemented in Table S1 and Table S2.

## Discussion

Our analysis of retrospective data found that the use of singleton birth weight percentiles resulted in a higher prevalence of SGA twins compared with the use of twin birth weight percentiles. Both of SGA twins defined by singleton and twin birth weight standards were associated with common morbidities such as neonatal unit admission, neonatal jaundice and ventilator support. In addition, SGA twins defined by twin standards were associated with BPD and the severe composite outcome whereas those defined by singleton standards were not.

The decision-making of clinical management on twin gestations depends on the chorionicity and the fetal growth [26–29]. Some clinicians identify abnormal growth among twins by using weight discordance, which has been proved to be associated with neonatal morbidity and be detected prenatally by ultrasound [30–32], although using discordant weight as the indication for delivery is still controversial. Using birth or estimated weight percentile is an effective way to identify growth-restricted fetuses. The prevalence of SGA twins depends on the definition used. Due to the lack of reference, growth standards for singletons are commonly used to assess twin growth in clinical practice [23, 33]. Previous studies showed that the growth trajectory of twins diverges from the one of singletons in the third trimester [13, 19, 34, 35]. Based on current study population, the use of singleton birth weight standards obtained a prevalence of 33.1 % in SGA newborns, which was nearly 3.5-fold higher than that by using twin standards. Similarly, the previous data from Houston, Texas [36], reported that the use of singleton growth standards misclassified twins as SGA, resulting in a 7-fold higher incidence of SGA fetuses compared with the use of twin growth standards. Also, another study of Nowacka et al. [37] showed the implementation of twin-specific nomograms decreased the rate of SGA twins, in comparison to using singleton-specific nomograms. The misdiagnosis of SGA could result in subsequent mistakes in clinical decision-making on the timing of delivery of SGA twin pregnancies.

Despite several birth weight standards for twin gestations has been published in recent years in China [15, 33, 38], these standards seem not promoted. We undertook a local validation to assess the appropriateness of the chosen standards. The analysis of neonatal outcomes showed that SGA twin, defined by either singleton or twin standards, were associated with significant increases in neonatal unit admission, neonatal jaundice and ventilator support. Based on the multiple analyses, these risks of morbidities are noted to be higher when using the twin standards than the singleton standards. The results were similar to those of sensitivity analysis performed among health women. Moreover, the twin standards showed an advantage for identifying newborns who were at risks of BPD, neonatal death as well as the composite severe outcome. These findings were consistent with that of previous studies [36, 37, 39]. Mendez-Figueroa et al. [36] found increased rates of composite morbidity, Apgar score < 4 at 5 min, mechanical ventilation, seizures, stillbirth as well as neonatal death in SGA fetuses defined by twin monogram whereas only increased rates of composite morbidity and stillbirth in SGA fetuses defined by singleton monogram [36]. Nowaka et al. [37] also found that estimating twin growth with customized charts provides better prognosis of adverse neonatal outcomes in the SGA twins compared with singleton nomograms. Giorgione et al. [39] found that twin charts identified a higher percentage of adverse outcomes (perinatal death, neonatal unit admission, preterm birth < 34 weeks and HDP) among SGA newborns than singleton charts. The twin birth weight percentiles were more applicable for identifying neonatal morbidities and mortality among twins. In this regard, the use of twin birth weight percentiles should be proposed and promoted in the clinical practice.

While the strengths in current study were large size of study population, several limitations should be acknowledged in the interpretation of the current results. First, some potential for bias could not be avoided due to the retrospective nature. Data regarding some potential confounders or neonatal outcomes (for example, maternal BMI, Apgar score) was unavailable or incomplete. Second, the chosen birth weight standards derived from Chinese population and therefore the conclusion might not be applicable to populations in other countries [15]. Third, sonographic EFW was not available in our retrospective database, therefore generalization of the current results to EFW deserves to be further studied.

## Conclusions

The use of singleton birth weight percentiles among twins contributes to higher prevalence of SGA newborns. The twin standards are more applicable for the prediction of adverse neonatal outcomes among twins. The use of the twin birth weight references in twin gestations should be proposed and promoted in clinical practice.

## Supplementary information

**Additional file 1.**

## Data Availability

The datasets used or analyzed in current study are available from the corresponding author on reasonable requests.

## Abbreviations

ACOG: American College of Obstetricians and Gynecologist
ART: assisted reproductive technology
DCDA: dichorionic-diamnionic
EFW: estimated fetal weight
FGR: fetal growth restriction
MCDA: monochorionic-diamnionic
GEEs: general estimated equations
OGTT: glucose tolerance test
GDM: gestational diabetes mellitus
HDP: hypertensive disorder of pregnancy
ICP: idiopathic jaundice of pregnancy
PE: pre-eclampsia
SGA: small for gestational age
TTTS: twin-to-twin transfusion
BPD: bronchopulmonary dysplasia
HIE: hypoxic ischemic encephalopathy
NEC: necrotizing enterocolitis
ICH: intracranial hemorrhage
NRDS: neonatal respiratory distress syndrome
