# Supplementary material for: Should singleton birth weight standards be applied to identify small-for-gestational age twins?: analysis of a retrospective cohort study
Source: BMC Pregnancy Childbirth. 2021 Jun 25;21:446. doi: 10.1186/s12884-021-03907-1 (PMC8234673; doi:10.1186/s12884-021-03907-1)
Supplement: Supplementary file 1 — Additional file 1. [file 12884_2021_3907_MOESM1_ESM.docx]

| Table S1. Analysis of association between SGA twin newborns and neonatal outcomes based on singleton birthweight reference among healthy women (n=3836). | | | | | | |
| --- | --- | --- | --- | --- | --- | --- |
| Outcomes | Non-SGA  (n= 2555) | SGA  (n= 1281) | Unadjusted OR (95% CI) | *P*-value | Adjusted OR (95% CI)^*^ | *P*-value |
| Neonatal unit admission | 975 (38.2) | 517 (40.4) | 1.26 (1.12-1.41) | <0.001 | 1.98 (1.67-2.35) | <0.001 |
| Neonatal jaundice | 716 (28) | 364 (28.4) | 1.18 (1.04-1.34) | 0.012 | 1.84 (1.54-2.20) | <0.001 |
| NRDS | 252 (9.9) | 71 (5.5) | 0.74 (0.61-0.88) | 0.001 | 0.99 (0.72-1.35) | 0.941 |
| Neonatal asphyxia | 63 (2.5) | 18 (1.4) | 0.66 (0.41-1.05) | 0.078 | 0.97 (0.55-1.70) | 0.909 |
| Ventilator support | 215 (8.4) | 70 (5.5) | 0.78 (0.62-0.98) | 0.037 | 1.44 (1.02-2.04) | 0.039 |
| HIE | 9 (0.4) | 3 (0.2) | 0.67 (0.18-2.46) | 0.541 | 0.79 (0.21-3.03) | 0.732 |
| ICH | 15 (0.6) | 4 (0.3) | 0.52 (0.17-1.60) | 0.256 | 0.70 (0.22-2.19) | 0.537 |
| Sepsis | 38 (1.5) | 9 (0.7) | 0.48 (0.23-1.00) | 0.050 | 0.70 (0.32-1.52) | 0.369 |
| BPD | 28 (1.1) | 6 (0.5) | 0.43 (0.18-1.05) | 0.063 | 0.98 (0.38-2.53) | 0.964 |
| Neonatal death | 12 (0.5) | 4 (0.3) | 0.60 (0.19-1.93) | 0.396 | 1.03 (0.31-3.38) | 0.965 |
| Severe composite outcome | 63 (2.5) | 12 (0.9) | 0.43 (0.23-0.77) | 0.005 | 0.71 (0.36-1.41) | 0.331 |
| ^*^, Adjusted for gestational age at delivery, nulliparity, maternal age, chorionicity, use of ART. NRDS, neonatal respiratory distress syndrome; HIE, hypoxic ischemic encephalopathy; ICH, intracranial hemorrhage; BPD, bronchopulmonary dysplasia; OR, odds ratio; ART, assisted reproductive technology. | | | | | | |

| Table S2. Analysis of association between SGA twin newborns and neonatal outcomes based on twin birthweight reference among healthy women (n=3836). | | | | | | |
| --- | --- | --- | --- | --- | --- | --- |
| Outcomes | Non-SGA  (n= 3592) | SGA  (n= 244) | Unadjusted OR (95% CI) | *P*-value | Adjusted OR (95% CI)^*^ | *P*-value |
| Neonatal unit admission | 1312 (36.5) | 180 (73.8) | 3.25 (2.63-4.02) | <0.001 | 6.32 (4.61-8.65) | <0.001 |
| Neonatal jaundice | 947 (26.4) | 133 (54.5) | 2.50 (2.02-3.11) | <0.001 | 4.00 (3.00-5.33) | <0.001 |
| NRDS | 288 (8.0) | 35 (14.3) | 1.08 (0.80-1.45) | 0.615 | 1.41 (0.88-2.25) | 0.151 |
| Neonatal asphyxia | 73 (2.0) | 8 (3.3) | 1.44 (0.73-2.82) | 0.294 | 1.68 (0.77-3.64) | 0.190 |
| Ventilator support | 252 (7.0) | 33 (13.5) | 1.52 (1.08-2.14) | 0.016 | 2.35 (1.44-3.83) | 0.001 |
| HIE | 10 (0.3) | 2 (0.8) | 2.95 (0.64-13.57) | 0.164 | 3.46 (0.74-16.14) | 0.113 |
| ICH | 17 (0.5) | 2 (0.8) | 1.91 (0.46-7.86) | 0.371 | 1.82 (0.41-7.97) | 0.429 |
| Sepsis | 43 (1.2) | 4 (1.6) | 1.27 (0.45-3.60) | 0.655 | 1.18 (0.42-3.33) | 0.760 |
| BPD | 29 (0.8) | 5 (2.1) | 2.48 (0.94-6.53) | 0.067 | 2.79 (0.98-7.92) | 0.054 |
| Neonatal death | 13 (0.4) | 3 (1.2) | 3.42 (0.98-11.93) | 0.054 | 3.59 (0.99-13.03) | 0.052 |
| Severe composite outcome | 69 (1.9) | 6 (2.5) | 1.18 (0.51-2.73) | 0.694 | 1.18 (0.49-2.87) | 0.715 |
| ^*^, Adjusted for gestational age at delivery, nulliparity, maternal age, chorionicity, use of ART. NRDS, neonatal respiratory distress syndrome; HIE, hypoxic ischemic encephalopathy; ICH, intracranial hemorrhage; BPD, bronchopulmonary dysplasia; OR, odds ratio; ART, assisted reproductive technology. | | | | | | |
